# The 4th Dimension in Animal Movement: The Effect of Temporal Resolution and Landscape Configuration in Habitat‐Selection Analyses

**DOI:** 10.1002/ece3.71434

**Published:** 2025-05-12

**Authors:** Johannes Signer, Cédric Scherer, Viktoriia Radchuk, Carolin Scholz, Florian Jeltsch, Stephanie Kramer‐Schadt

**Affiliations:** ^1^ Wildlife Sciences, Faculty for Forestry and Forest Ecology University of Goettingen Göttingen Germany; ^2^ Department of Ecological Dynamics Leibniz Institute for Zoo and Wildlife Research Berlin Germany; ^3^ Institute of Biochemistry and Biology University of Potsdam Potsdam Germany; ^4^ Institute of Ecology Technische Universität Berlin Berlin Germany

**Keywords:** animal movement, habitat selection, parameter estimation, resource‐selection function, sampling interval, sensitivity, step‐selection function, telemetry

## Abstract

Understanding how animals use their habitat is essential to understand their biology and support conservation efforts. Technological advances in tracking technologies allow us to follow animals at increasingly fine temporal resolutions. Yet, how tracking devices' sampling intervals impact results remains unclear, as well as which method to use. Using simulations and empirical data from wild boars tracked in Germany, we systematically examine how the temporal resolution of movement data in interaction with the spatial autocorrelation of the landscape affects the outcomes of two common techniques for analyzing habitat selection: resource‐selection analysis (RSA) and an autocorrelation‐informed weighted derivative (wRSA) as well as integrated step‐selection analysis (iSSA). Each method differs in the definition of “available” locations (RSA) and the implementation of the movement model during parameter estimation (iSSA). Our simulations suggested that landscape autocorrelation has a much stronger effect on the estimated selection coefficients and their variability than the sampling interval. Higher sampling intervals (i.e., longer time between steps) are required for landscapes with high autocorrelation, enabling the animal to experience enough variability in clumped landscapes. Short sampling intervals generally lead to higher variability and fewer statistically significant estimates (in particular for wRSA). Our results complement recent attempts to outline a coherent framework for habitat‐selection analyses and to explain them to practitioners. We further contribute to these efforts by assessing the sensitivity of two commonly used methods, RSA and iSSA, to the changes in sampling interval of movement data. We expect our findings to further raise awareness of pitfalls underlying the comparison of estimated selection coefficients obtained in different studies and to assist movement ecologists in choosing the appropriate method for habitat‐selection analysis.

## Graphical abstract

Advances in tracking technology enable finer temporal resolution in animal movement data, but the impact of sampling intervals on habitat‐selection analysis remains unclear. Using simulations and wild boar data from Germany, researchers found that higher sampling intervals are needed in highly autocorrelated landscapes to capture habitat variability, while shorter intervals result in greater variability and fewer significant estimates. These findings contribute to a broader framework for improving habitat‐selection analyses and guiding practitioners.

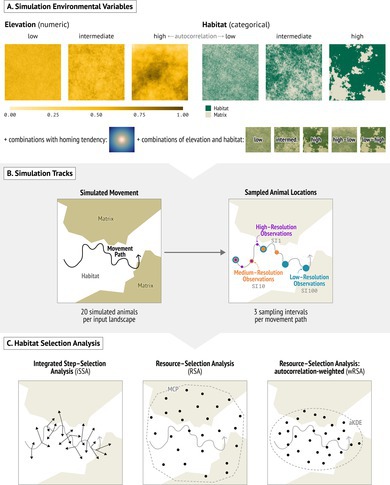

## 1 Introduction

The last decade has brought great progress in animal telemetry (Kays et al. 2015; Nathan et al. 2022). Obtaining high‐quality movement data is nowadays possible for many vertebrate species and provides detailed insights into the behaviors of wild animals and their consequences to individual fitness and population dynamics (Hertel et al. 2020; Nathan et al. 2022). A fundamental question in ecology and conservation that movement data informs is the choice of habitat features by individuals (Northrup et al. 2022). It allows us to better understand the space use of animals (McNit et al. 2020), to evaluate reasons for human‐wildlife conflicts (Takahata et al. 2013), and to design appropriate conservation measures such as the placement of movement and dispersal corridors (e.g., Bastille‐Rousseau and Wittemyer 2021; Van Moorter et al. 2021).

The most common approaches to assess habitat selection based on movement data are resource‐selection analysis (RSA) and step‐selection analysis (SSA). The origin of both methods is the resource‐selection function (RSF) proposed by Manly et al. (1993), which was disseminated by Boyce and colleagues (Boyce et al. 2002; Boyce and McDonald 1999) and later extended to the step‐selection function (Avgar et al. 2016; Forester et al. 2009; Fortin et al. 2005). The objective of both approaches is to estimate the selection behavior of animals with respect to habitat variables. More precisely, RSA and SSA estimate parameters of a function that models the probability of selecting a location, if available, based on its environmental characteristics, relative to other locations (with different environmental features). The basic methodological concept is to statistically contrast observed animal locations (representing used habitat) with the landscape that is available (i.e., habitat that could potentially be used), which is typically represented by a random sample of locations from an area that is deemed within animals' reach. The estimated parameters are then interpreted in terms of animal behavior, i.e., preference or avoidance of certain environmental features relative to others (Avgar et al. 2017; Fieberg et al. 2021) or used to generate predictions about animal space use (e.g., Fischer et al. 2019; Overton and Casazza 2023) and connectivity (Hooker et al. 2021; Meyer et al. 2020).

The key difference between RSA and SSA is their assumption about the availability of habitat, and inherently linked with this are assumptions about the data‐generating process. RSA assumes a static domain of availability, like a home range, and that observed animal locations are independent samples of this domain, constrained only by animals' selection behavior (Fieberg et al. 2010). This assumption is acceptable for low‐resolution data but often violated for contemporary high‐resolution movement data, since animals are limited in their movement distance between consecutive location fixes (Fieberg et al. 2010; Northrup et al. 2022). One option is to thin high‐resolution movement data prior to analysis (Northrup et al. 2013; Stillfried et al. 2017); however, this practice is debatable, as it not only wastes data, but may also remove biological signal (Dormann 2007). More recently, it was suggested to explicitly model temporal autocorrelation in RSA by adding autocorrelation‐informed weights using continuous‐time movement models (Alston et al. 2022).

Along a different vein, SSA was developed to alleviate the problem by defining availability conditional on the current position of the animal through a movement model with an explicit time dimension that accounts for correlation between subsequently observed locations (Forester et al. 2009). For SSA, steps are assumed to be independent and not positions as with RSA. This has the added benefit of allowing the investigation of a wider variety of behavioral processes than mere resource selection in the original sense. Examples of such behavioral processes are cognitive mechanisms of space use (e.g., Oliveira‐Santos et al. 2016; Schlägel et al. 2017) and interactions between animals (Potts et al. 2014; Schlägel et al. 2019).

However, the more explicit consideration of the movement process in SSA reveals a possible new dilemma regarding the sampling interval of movement data. Movement data used for RSA and SSA originate from automated telemetry systems (e.g., GPS, automated radio‐telemetry systems, or reverse‐GPS systems). A critical feature of such data is the time interval between consecutive locations, i.e., the temporal resolution or sampling interval of observed movement paths. As battery power of animal‐borne transmitters is limited and strongly depends on the body mass of study species (Wilson et al. 2021), sampling intervals are often chosen to balance total deployment time and temporal resolution according to the biological question (Breed et al. 2011). This leads to highly variable temporal resolutions of data sets across studies. However, it is well known that inference from movement data can be sensitive to the data resolution (e.g., Postlethwaite and Dennis 2013; Rowcliffe et al. 2012). In particular, inference based on discrete‐time movement models (which applies for RSA and SSA) is typically sensitive to changes in temporal resolution, i.e., the assumed time interval between regular GPS fixes (Codling and Hill 2005; Mills et al. 2006; Ryan et al. 2004; Schlägel and Lewis 2016a). This poses two challenges: First, it complicates the comparison of studies based on different sampling schemes. Second, we typically do not know the appropriate temporal resolution that matches the scale of biological decision processes studied. In addition, the time scales for behavioral decisions likely change themselves in relation to, e.g., behavioral mode. Simply imposing a temporal resolution based on technical reasons during data collection may then yield misleading results and ultimately affect decision‐making (e.g., in conservation).

Another issue interwoven in the temporal resolution of movement data is that of spatial autocorrelation of landscape covariates influencing the sensitivity of selection coefficients (Avgar et al. 2016). To better understand factors that may influence our inference from habitat selection studies, we studied the effect of temporal resolution and landscape configuration in RSA and SSA analyses in concert. We expected the subtle, yet meaningful, difference between RSA and SSA to impact their sensitivity to temporal resolution. The movement model underlying SSA makes inference potentially sensitive to the time interval between consecutive locations. For example, analysis of a one‐dimensional version of the step‐selection model demonstrated that estimated selection strength increased with increasing sampling interval (Schlägel and Lewis 2016b) for the following reason: At extremely fine scales, there is very little environmental variation between steps, corresponding to weaker selection. Likewise, at larger time intervals, selection estimates are seemingly stronger due to more environmental variation at larger spatial scales. This also implies that the larger the time interval, the more similar estimates from RSA and SSA will be. In contrast, since RSA explicitly considers neither time nor movement, there is no clear indication of an effect of sampling interval on inference results. However, decreasing the sampling interval may lead to spatial autocorrelation in the data, because an animal is less likely to travel into a different habitat type within a smaller time interval. Furthermore, the effective amount of information of the data will decrease as the sampling interval increases (with a constant number of relocations; Fleming et al. 2019). Although omitting spatial autocorrelation typically causes more concerns about deflated standard errors than biased parameter estimates (Fieberg et al. 2010), a study of a related method of species distribution modeling found biased parameter estimates in models ignoring spatial autocorrelation (Dormann 2007). Therefore, we also include RSA in our evaluation of sensitivity to temporal resolution and compare it to SSA.

For RSA, we implemented the original approach of sampling available points as random points uniformly from the availability domain (Boyce et al. 2002; Manly et al. 1993). However, distributing random points assumes that the animal can cross its entire home range between two relocations, which is often unrealistic. We assume this effect to be important only for small sample sizes, i.e., when only a small number of relocations were collected. We additionally implemented autocorrelation‐informed weighting to mitigate pseudoreplication in high‐resolution movement data that are inherently autocorrelated (wRSA; Alston et al. 2022) as a second method.

For SSA, we implemented integrated step‐selection analysis (iSSA; Avgar et al. 2016) that simultaneously models movement and habitat selection of the animal. To fit this model, each observed step (straight line between consecutive observations) is paired with random control steps, and a conditional logistic regression is used to obtain parameter estimates. This approach can be formally derived as an approximation to fitting a random‐walk movement model in which movement decisions of an animal are influenced by an RSF (Forester et al. 2009).

Our goal here is to investigate how sensitive different analytical approaches are to different temporal resolutions of tracking data, while considering the constraints of collar battery power by keeping the maximum number of fixes constant. For our analysis, we used both simulated data and empirical GPS data of wild boar ( *Sus scrofa* ) from Berlin, Germany. We investigated how parameter estimates pertaining to habitat selection varied with changing temporal resolution of tracking data and various degrees of spatial autocorrelation in the underlying landscapes to contribute to a coherent framework. Additionally, we investigated how changing temporal resolution affected differences in predictive space‐use maps and thus potentially may confound decision‐making in conservation management and protected‐area design.

## 2 Materials and Methods

### 2.1 Data

#### 2.1.1 Simulated Data

To explore the effect of sampling interval and landscape configuration on the estimated selection coefficients, we conducted a simulation study in discrete space and time. We simulated 10 realizations of a landscape with three covariates (a discrete and a continuous covariate, as well as distance to home range center) and applied three different habitat‐selection analyses (two versions of RSA and SSA) to each realization (see Section 2.2 for details).

**FIGURE 1:**
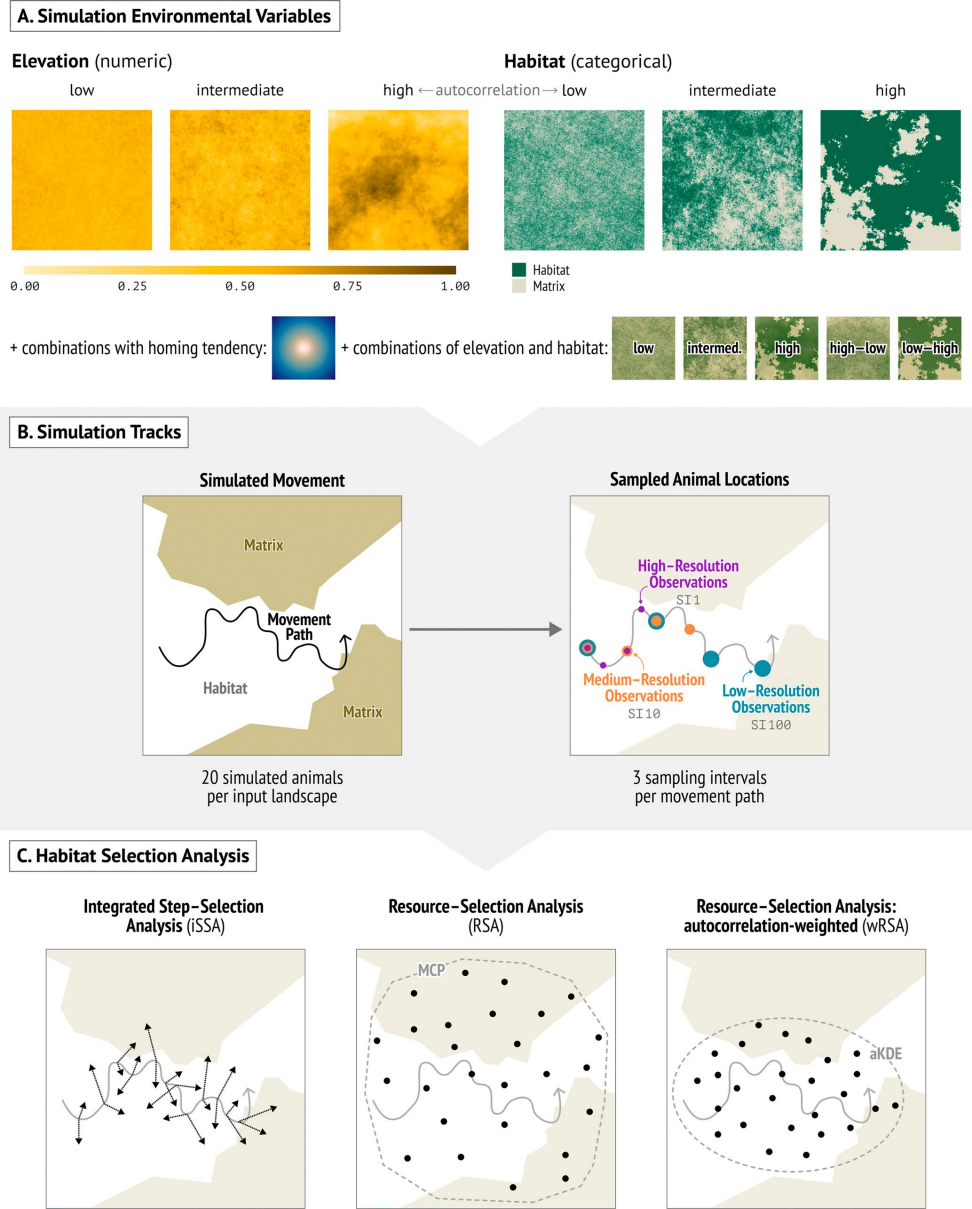
Schematic of the implemented analytical methods and simulation study of scenario 1. (A) For each of the replicated landscape settings with different spatial autocorrelation and their combinations, we (B) simulated 20 repetitions of nearly‐continuous animal movement tracks influenced by these variables and subsampled the tracks to three different sampling intervals. (C) The tracks of different intervals were analyzed with three method variants (iSSA, RSA, and wRSA). Multicollinearity assessment of the combined landscape scenarios is provided in Figure S2.

We created 10 replicates of landscapes (2000 by 2000 raster cells) with three layers of environmental predictor variables: a binary variable indicating a habitat feature (*hab*), like a forest, grassland, or urban area, and a continuous variable representing, for example, elevation (*elev*) (Figure 1A, Figure S1). We simulated *hab* and *elev* as two‐dimensional fractional Brownian motion using the R‐package NLMR (Sciaini et al. 2018; Travis and Dytham 2004) with three different values for the fractional dimension: 0.01, 0.1, and 1 corresponding to no, intermediate, and high landscape autocorrelation, with covariate values ranging between 0 and 1 (Figure S1). Furthermore, we categorized *hab* into a binary variable by setting values greater than 0.5 to 1 (habitat feature present) and all others to 0 (habitat feature absent), resulting in approximately 50% of each landscape consisting of a certain habitat feature.

Using these landscapes, we simulated time‐discrete animal movement with a stepping‐stone process (see Signer et al. 2017 for details on the algorithm). In brief, at each time step, an animal moves away from the currently occupied raster cell with probability 0.3, and if it moves, it chooses one of the four cells in the von Neumann neighborhood based on its preferences for the two environmental variables *elev* and *hab* described above. In order to obtain realistic movement tracks, we rarefied relocations by retaining only every 100th point at the largest sampling interval. We simulated animals to avoid the habitat feature (*ω*_hab_ = −2) and preference for higher elevation (*ω*_elev_ = 2). As many animals often move within restricted home ranges, we further simulated animals to have a centralizing or homing tendency. This was achieved by including a third auxiliary environmental covariate “distance to home range center” (non‐standardized; preference parameter *ω*_cent_ = −0.01), with the home range center placed at the center of each landscape. Each simulated movement path started at a random location within a centered square with coordinates 800, 800, and 1200, 1200. Note, we simulated with and without the centralizing tendency. If we included a centralizing tendency, the variable was not included as an explanatory variable in the resource‐selection and step‐selection models.

Movement paths of individual animals were simulated for 10,000,000 time steps and rarefied to every 100th time step during the simulations. To analyze the data, we further rarefied by 1 (high temporal resolution or short sampling interval, small time difference between relocations or small time interval), 10, or 100 (low temporal resolution or large sampling interval, large time difference between relocations) time units (i.e., movement steps; Figure 1B). For each rarefaction interval, we always used the 1000 first relocations to ensure the same number of data points, resembling battery lifetime. In biological terms, this may, for example, correspond to sampling intervals ranging from 10 min to ca. 16 h. For each of the 10 replicated landscape sets, we simulated 20 movement tracks, resulting in a total of 18.000 movement track replicates (10 landscapes × 20 individuals × 3 landscape configurations, with and without home ranging, inclusion of 1 or 2 covariates, three sampling/rarefaction rates).

#### 2.1.2 Empirical Data

**FIGURE 2:**
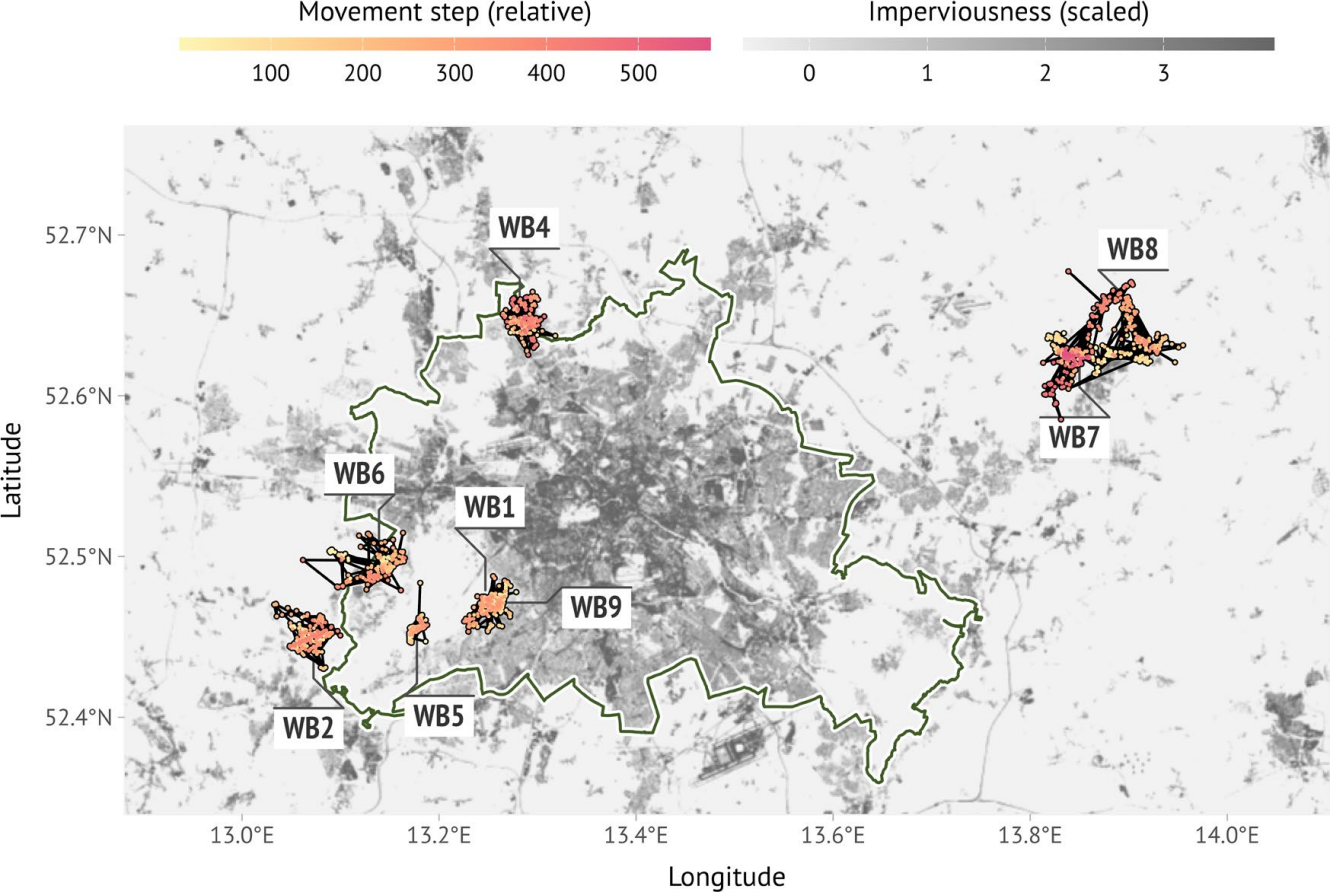
Location of the tracks of eight wild boar (numbers stand for IDs) within the borders of Berlin, Germany, and surroundings. The points sampled at 12 h intervals are shown. The greyscale gives the proportion of sealed surface in a 100 × 100 m grid (dark gray: Highly sealed).

We used high‐resolution movement data of urban and rural wild boar ( *Sus scrofa* ) in the capital city Berlin and the federal state of Brandenburg, Germany (Stillfried et al. 2017) (Figure 2). Wild boar were tracked with 30 min intervals between 2013 and 2015, which we considered a high resolution given that they rest and hide most of the time during the day, and move about a maximum of 100 m in 30 min during peak time (e.g., Quy et al. 2014); hence, 30 min is a small interval in relation to the movement distances covered. Smaller intervals would have led to a lot of effective zero distances, which would realistically only mean unnecessary errors due to GPS errors and no real movements (Jerde and Visscher 2005).

Mean observation time of collared wild boar was around 6 months. We only used data from wild boar that had > 150 locations left at the lowest temporal resolution after rarefaction, which resulted in movement data from eight out of the originally tracked 11 wild boars. The GPS tracks were subsampled to four different sampling intervals of 60 min, 360 min (6 h), 720 min (12 h), and 1440 min (24 h) (Table S1).

The study area is characterized by rural areas composed of small forests mostly embedded in agricultural landscapes, with up to 73% of arable land. The urban environment within our study area reaches values of imperviousness up to 100% in the city center, whereas in total 20% of the urban fabric is forested.

We derived three variable types analogously to the simulation study in a raster of 100 × 100 m: imperviousness (continuous), forest habitat (categorical based on tree cover > 75%), and distance to water (continuous) (Figure S6). 100 × 100 m raster grids for imperviousness and forest habitat were derived from 20 × 20 m (original raster resolution) Copernicus data from 2015 (www.copernicus.eu). We calculated the distance to linear water elements like rivers and water bodies such as lakes based on maps provided by the federal states of Berlin and Brandenburg. The Berlin map is named “Umweltatlas Berlin/Stadtstruktur—Flächentypen differenziert 2010 (Umweltatlas)” and was downloaded from the FIS‐Broker data portal (www.fisbroker.de). The Brandenburg map is named “Kartierung von Biotopen, gesetzlich geschützten Biotopen (§ 30 BNatSchG und § 18 BbgNatSchAG) und FFH‐Lebensraumtypen im Land Brandenburg” and the source is www.mlul.brandenburg.de/lua/gis/biotope_lrt.zip.

### 2.2 Habitat Selection Analysis

#### 2.2.1 Resource‐Selection Analysis

For RSA, we used the classic approach that fits a logistic regression model to “used” and “available” locations (Manly et al. 1993). This was implemented through a generalized linear model (GLM) with binomial error distribution and logit link function. The result of this method yields estimates for the selection parameter $\beta =\left(\beta_{1},\ldots ,\beta_{m}\right)$ relating to *m* environmental predictor variables in the exponential form of the RSF

(1) $$w\left(z(x\right);\beta )=\exp\left(\beta_{1}z_{1}\left(x\right)+\ldots +\beta_{m}z_{m}\left(x\right)\right),$$

where $z\left(x\right)=\left(z_{1}\left(x\right),\ldots ,z_{m}\left(x\right)\right)$ contains the values of predictor variables taken at location x. The method assumes that all observations are independent of each other. Note that selection parameters of this model are related to, but not necessarily equal to the *ω* values that we used in the simulation above due to the likely scale‐dependency of parameters.

To sample available locations from this area, we sampled random points uniformly in space (implemented in the R‐package *amt*; Signer et al. 2019; Figure 1C), sampling 20 times as many available locations as used locations given by the animal path. As a second RSA method, we used autocorrelation‐informed weighting of points (Alston et al. 2022) using the R‐package *ctmm* (Calabrese et al. 2016). This method weights each observed location in an animal's movement track according to its level of non‐independence. We first fitted a semi‐variogram and an aKDE home range from the movement data and estimated resource selection as described in Alston et al. (2022).

#### 2.2.2 Step‐Selection Analysis

The intuitive approach to iSSA is to modify RSA such that steps (the directional pair of two consecutive locations) become the unit of interest, and the contrast between used and available steps occurs at the scale of individual steps. That is, for each observed step, one samples a set of available steps, starting at the same location as the observed step and considering movement capacities of the animal given by step length (SL) and turning angle (TA) (Figure 1C). Each observed step, together with its corresponding available steps, constitutes a stratum. The stratified data is accounted for in the analysis by fitting a conditional logistic regression to obtain estimates for Equation (1).

iSSA can be formally derived from a random‐walk movement model, in which the probability of making a step from one location to the next is composed of a movement kernel (specifying how the animal would move if it had no specific attitudes (preferences/avoidance) toward habitat features) and a habitat‐selection function, which is modeled after an RSF (Forester et al. 2009). More precisely, the probability of making a step from location $x_{t-1}$ to location $x_{t}$ within the (constant) sampling interval $\left[t-1,t\right]$ is given by

(2) $$p\left(x_{t}|x_{t-1},x_{t-2},\theta ,\beta \right)=\frac{\Phi \left(x_{t},x_{t-1},x_{t-2};\theta \right)\cdot w\left(z\left(x_{t}\right);\beta \right)}{\int_{y\in D}\Phi \left(y,x_{t-1},x_{t-2};\theta \right)\cdot w\left(z\left(y\right);\beta \right)\;\mathrm{dy}},$$

where Φ is the movement kernel (depending on parameter vector θ) and w is a selection function of the same form as the RSF in Equation (1). The previous location $x_{t-2}$ is included because Φ is typically based on turning angles, whose computation requires three consecutive locations. This model is commonly fitted with a conditional logistic regression and the integral in the denominator is approximated with random steps (Fortin et al. 2005; Forester et al. 2009; Avgar et al. 2016; Michelot et al. 2024).

We sampled 20 available steps per observed step based on a gamma distribution for SL and a von Mises distribution for TA, which were fitted to the observed data (Fieberg et al. 2021; Signer et al. 2019). Covariate values belonging to a step were extracted at the end location of a step. We then assessed estimated selection coefficients *β* of the fitted models for the single and combined environmental predictors (Figure 1C) and compared them to the input coefficients of the simulated tracks. All model fits were performed separately for individual movement paths, i.e., we did not pool data from multiple animals.

## 3 Results

### 3.1 Simulations

**FIGURE 3:**
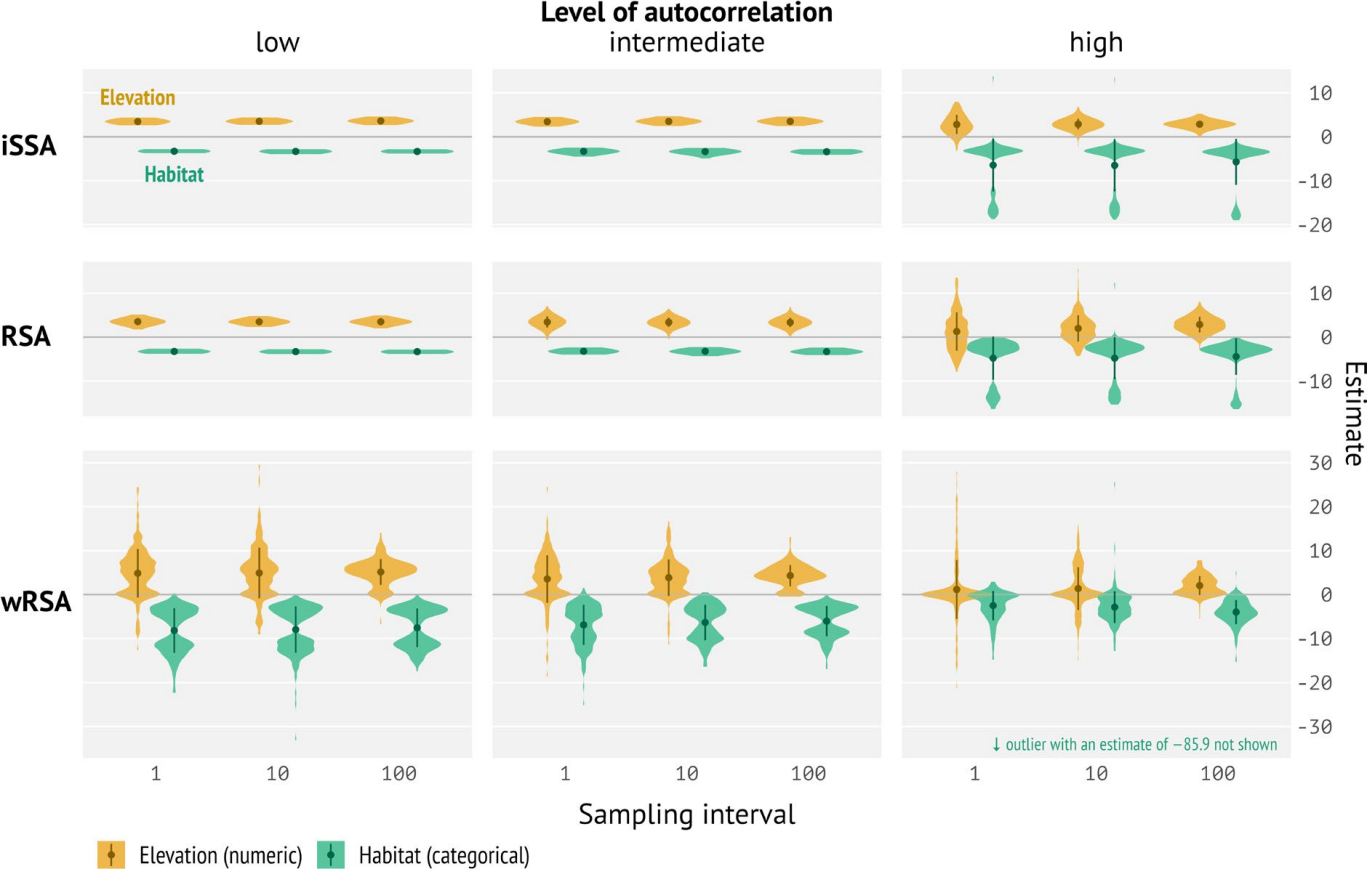
Distribution of selection parameters estimated by integrated step selection analysis (iSSA), resource‐selection analysis (RSA), and autocorrelation‐informed weighted RSA (wRSA) from 200 replications pooled over 10 landscapes for single landscape simulations and analyses. 1 means the smallest sampling interval (highest temporal resolution) and 100 the lowest (small temporal resolution). We varied the sampling intervals (*x*‐axis) and the autocorrelation in the landscape (columns). Note, we expect a positive coefficient for elevation (+2) and a negative coefficient (−2) for habitat feature. *X*‐axes are differently scaled.

**FIGURE 4:**
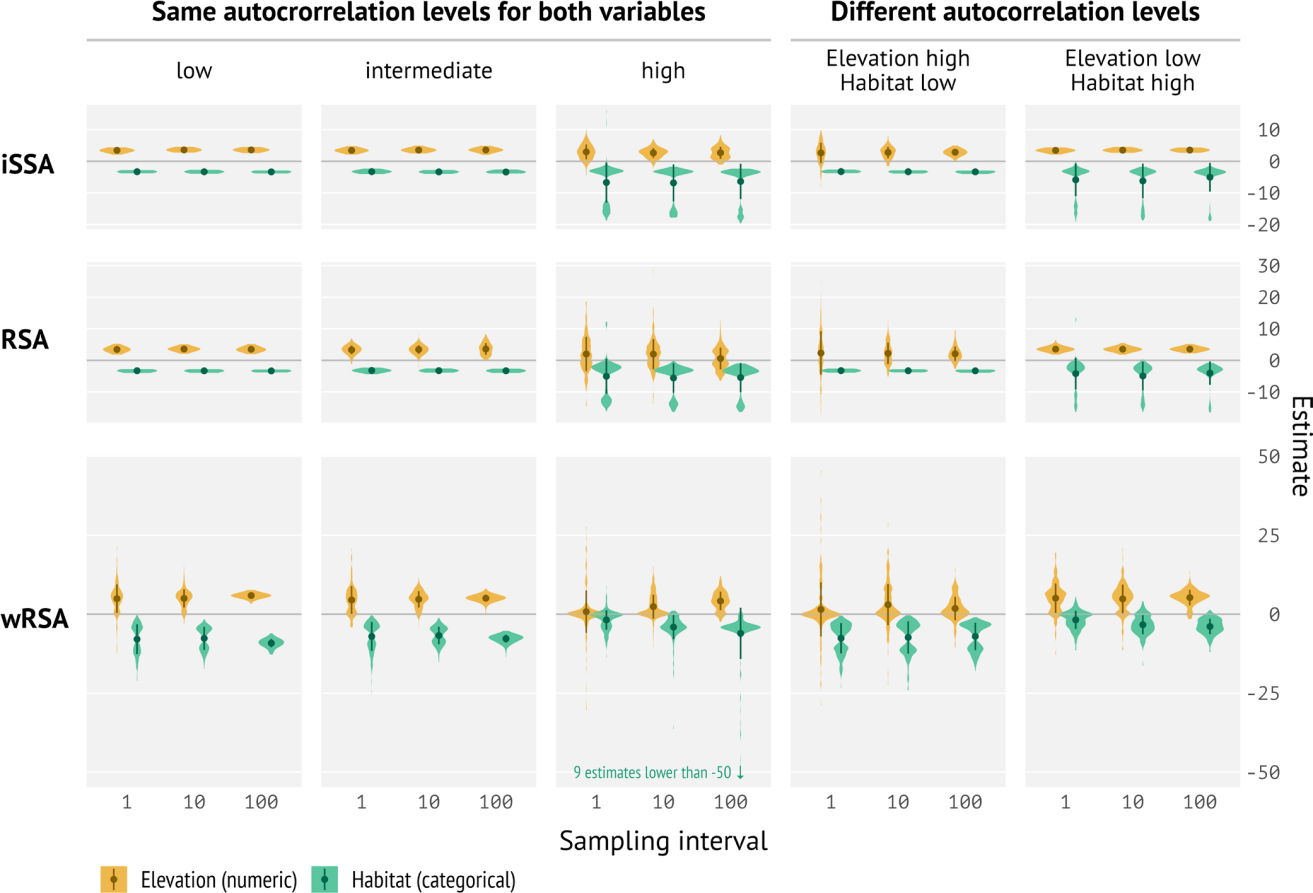
Distribution of selection parameters estimated by integrated step selection analysis (iSSA), resource‐selection analysis (RSA), and autocorrelation‐informed weighted RSA (wRSA) from 200 replications pooled over 10 landscapes for simulations and analyses with two covariates separately and combined. 1 means the smallest sampling interval (highest temporal resolution) and 100 the lowest (small temporal resolution). We varied the sampling intervals (*x*‐axis) and the autocorrelation in the landscape (columns). Note, we expect a positive coefficient for elevation (+2) and a negative coefficient (−2) for habitat feature. *X*‐axes are differently scaled.

Generally, home ranging stabilized results and led to less variation (Figure S5). We present here only results where home‐ranging behavior was included in the simulations, but not estimated in the models. Differences between the landscape realizations were minimal; we thus pooled across landscapes. Generally, we noted that the autocorrelation level of the landscape had a much higher impact on the results than the sampling interval (Figures 3 and 4, columns for the landscape autocorrelation and *x*‐axis for the sampling interval; Figures S3 and S4). We start by looking at simulations that included only one covariate. Little to no autocorrelation in the landscape yielded very stable estimated coefficients (Figure 3 column 1). As landscape autocorrelation increased, the variability of estimated covariates increased, particularly for RSA and iSSA (Figure 3, first and second row). For wRSA, the variability was generally higher. Larger sampling intervals (i.e., more time between consecutive positions) led to lower variability between replications. This was most obvious for landscapes with high autocorrelation (Figure 3, right column).

If both covariates (elevation and habitat) were used for simulating animal tracking data, were included in the analysis, and had the same amount of landscape autocorrelation (Figure 4, columns 1, 2, and 3), the results were very similar to the scenarios with only one covariate. Interestingly, if the autocorrelation was different for the two covariates, results were again very similar to the single‐variable scenarios (Figure 4, columns 4 and 5).

**FIGURE 5:**
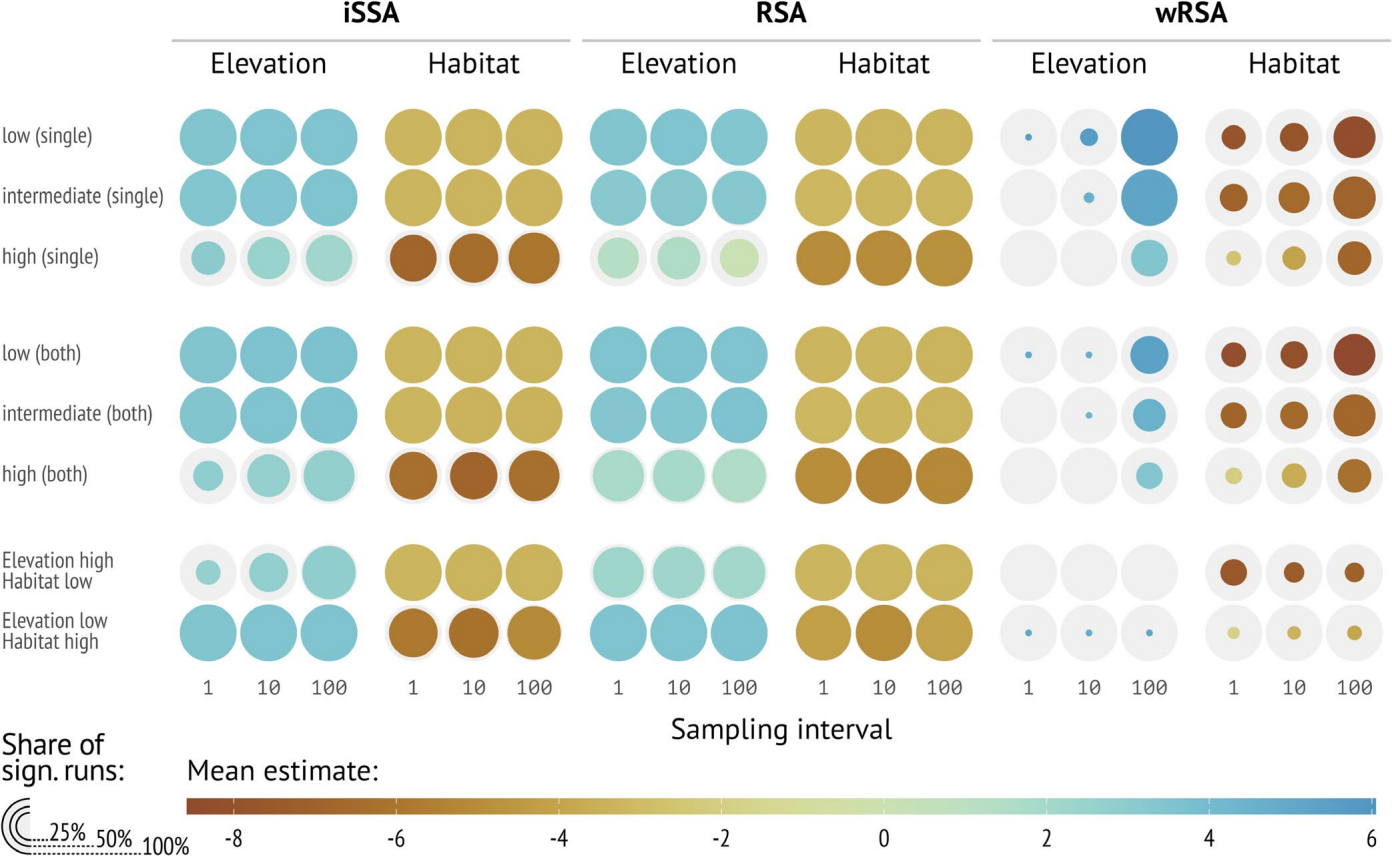
Fraction of estimated coefficients that are significantly different from 0 (given by the size of the colored dots), for different analysis methods (columns) and variable combinations (rows). We show three different sampling intervals (*x*‐axis), where 1 means the smallest time interval (highest temporal resolution or shortest sampling interval) and 100 the lowest, and different degrees and combinations of autocorrelation in the landscape (*y*‐axis). Note, we expect a positive coefficient for elevation (+2) and a negative coefficient (−2) for habitat feature.

We further looked at the fraction of significant estimates (checking if 95% confidence intervals overlapped 0 or not) for each analysis method as a function of landscape configuration and sampling interval. We observed similar patterns for RSA and iSSA: if the landscape autocorrelation was low to intermediate, we almost always observed that the estimated coefficients were significantly different from 0. This pattern was also consistent if we included both covariates in the simulations, if the covariate of interest had a low to intermediate autocorrelation of the landscape. If the autocorrelation was high, we observed that the fraction of significant results increased for coefficients estimated with iSSA, but was generally lower for RSA (Figure 5). For wRSA we found a stronger effect of the sampling interval on the fraction of significantly estimated coefficients, such that often at the smallest sampling interval (where the data were most autocorrelated), no significant effects were found, while at the largest sampling interval (less temporal autocorrelation of the data) the fraction of significant estimates was much higher.

### 3.2 Empirical Case Study

**FIGURE 6:**
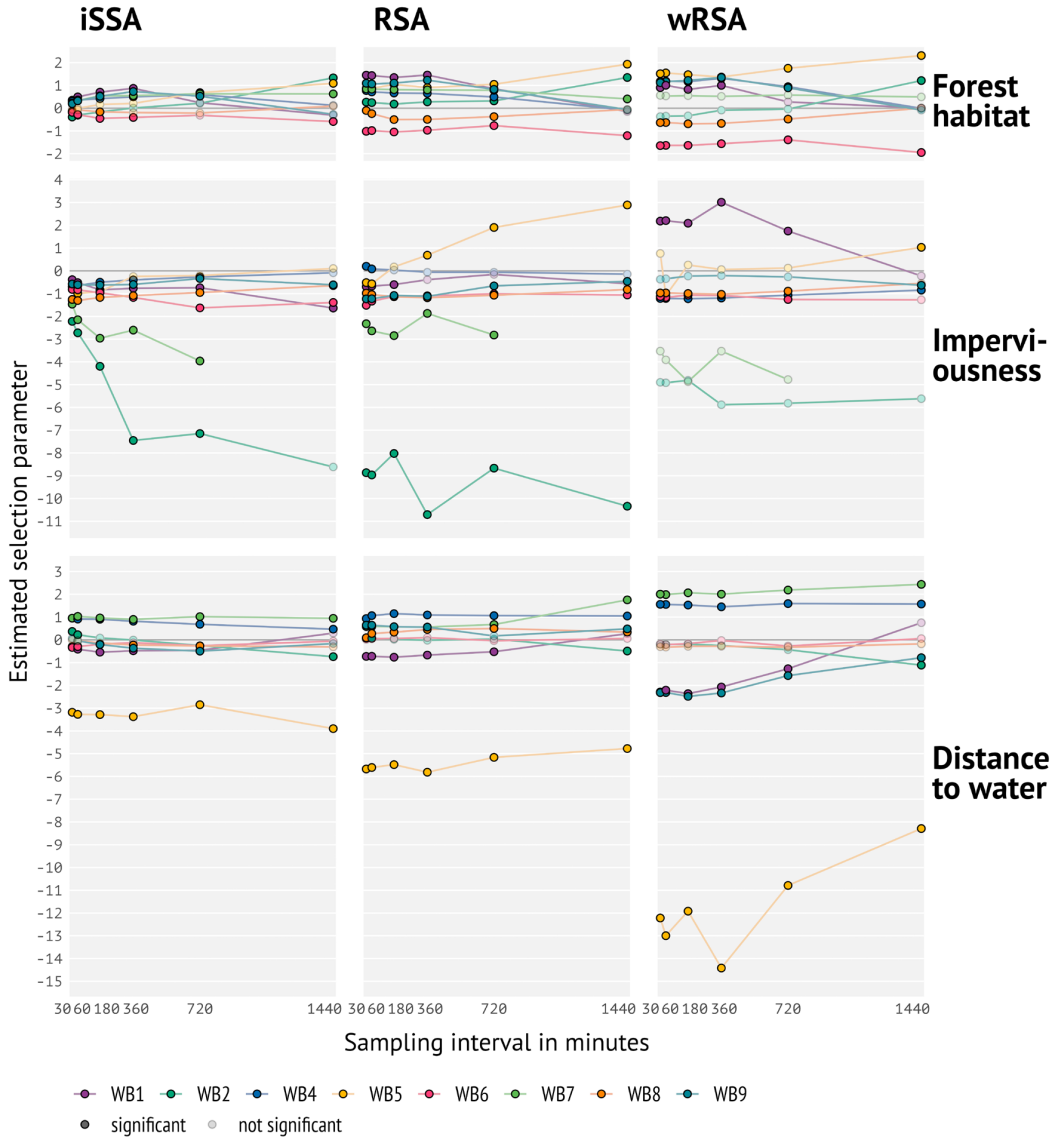
Selection parameters estimated by RSA, wRSA, and iSSA for the wild boar field data (numbering and colors to wild boar IDs; see also Figure 2). Transparency levels indicate if an estimated coefficient was significantly different from 0 (at an alpha level of 5%). Note that negative estimates with respect to “distance to water” indicate a preference toward water (selection for smaller distances).

Following results from the simulation scenarios, we focused on RSA, wRSA, and iSSA for the empirical data. In accordance with our simulation results, we found higher consistency in inter‐individual variability and less variation in selection estimates in iSSA for the eight tracked wild boar for environmental predictor variables ‘habitat type’ and ‘imperviousness’, although the pattern is less pronounced than in the simulations (Figure 6). This result is mirrored in the distributions of habitat features in the sampled available locations, iSSA‐based available locations better capturing the distribution from the observed data across temporal thinning than RSA‐based available locations (Figures S8–S10). RSA and wRSA showed high variability in estimates across wild boar individuals (e.g., wild boars WB 2, 5, and 7; Figure 6), and even sign switching for ‘imperviousness’ (WB 1, WB 5) and distance to water (WB 1) at large sampling intervals (note, that unlike with the simulation studies, for the empirical data the number of data points decreased as sample interval increased); i.e., changing from positive to negative values or vice versa, meaning going from avoidance to preference. Interestingly, at high temporal resolution, the estimates were much less variable iSSA than for RSA, especially for the environmental variables expressed by gradients like imperviousness and distance to water. Sign switching could most probably be an effect of the environmental variables that level themselves out, e.g., when wild boars seem to prefer high levels of imperviousness, because at the same time they have access to reed beds with high cover close to lake areas. This is, for example, the case of wild boars 2 and 5.

## 4 Discussion

The goal of this study was to understand the effects of landscape autocorrelation and movement data sampling interval on estimated selection strengths for RSA, wRSA, and iSSA, and to provide guidance to practitioners on which method to use. We used simulations to evaluate different methodological approaches to estimate selection coefficients. Overall, we found that parameter estimates were more sensitive to the autocorrelation of the landscape than to different sampling intervals. iSSA and RSA also showed lower variability than wRSA, which explicitly accounted for temporal autocorrelation in the data, whereas the former two do not. Temporal filtering (i.e., increasing the sampling interval) could correct for temporal autocorrelation of the telemetry data in spatially autocorrelated landscapes and thus also increase the amount of information that is contained in the data. We standardized the sample size in the simulations to 1000 regardless of the sampling interval to mimic battery lifetime, i.e., either having 1000 data points from short time intervals, e.g., several hours, or from larger intervals, e.g., days. However, it is important to note that 1000 relocations at the shortest sampling interval do not contain the same amount of information as 1000 relocations at a sampling interval that is 10 or 100 times coarser due to high autocorrelation in high‐resolution data. That is, if data are collected at high resolution, e.g., seconds or minutes (e.g., Grabow et al. 2024), the area covered will be very small, and there is not much variation in the landscape underlying the movement track.

RSA and iSSA do not account for temporal autocorrelation, which leads to similar results for landscapes with low to intermediate spatial autocorrelation, regardless of the sampling interval. Only for highly correlated landscapes do we see that the sampling interval becomes important. The reason for this is that for small sampling intervals, the variability in highly correlated landscapes is too small to detect a clear signal, and we see high variability in the estimates. In some instances, we even observed a sign switch of the selection coefficient.

In SSA, the direction of change in estimated selection strength varied with the type of environmental variable. For the categorical variable “habitat feature” (here, a binary variable distinguishing only presence and absence of that feature), estimated selection strength increased marginally with increasing sampling interval. This was likely due to the “clumping” of habitat within patches in the landscape. When using finer temporal data, availability (defined based on movement constraints at that time interval, like SL) is at a finer spatial scale as well, i.e., step lengths are also usually small. Hence, available (control) locations are also sampled at a finer spatial scale because, in a short time, animals move less far. In our study, this is more similar to the scale at which the simulated animals made movement decisions. If the individual is within a suitable habitat patch, available locations relatively often fall into the same patch. At coarser temporal scales or lower autocorrelation of the landscape, available locations are more variable, creating a stronger contrast between covariate values of used and available locations. Correspondingly, the comparison between used and available locations appears to be stronger. In addition, wRSA also required larger sampling intervals to find statistically significant results (Figure 6). This is a feature of wRSA because short sampling intervals lead to higher temporal autocorrelation of tracking data.

Selection strengths for landscapes with little to no autocorrelation were unaffected by the sampling interval. This suggests that models with multiple covariates may conceal individual effects in the combined landscape simulations. We found absolute values of selection strength were not affected or increased slightly with increasing sampling interval. This is good news, as this suggests that iSSA results do not vary as much across different sampling intervals as expected. On the other hand, it also suggests that data sets, such as the wild boar case study, may consistently lead to higher selection strength if animals' behavioral scale of decision‐making was finer than the tracking sampling scheme. In fact, this pattern is consistent with theoretical results on scale‐dependence in habitat‐selection analysis that indicate that RSA parameters will be roughly twice the SSA parameters (Moorcroft and Barnett 2008). Inferences from any SSA are tied to the temporal resolution. Similarly, modifying spatial autocorrelation can be viewed the same as changing temporal resolution (Avgar et al. 2016). Hence, knowing the “true” behavioral scale at which animals make their movement decisions is difficult (e.g., Roberts et al. 2017), and assessing this problem is beyond the scope of this study. This emphasizes that researchers need to be mindful if the data resolution matches the scale of their research questions.

Interestingly, RSA showed little to no sensitivity to the sampling interval, but strong sensitivity to the landscape setting. Or in other words, the RSA is more sensitive to landscape features outside the animal's home range (habitat selection order 2; Johnson 1980), while SSA is related to selection at the path level (habitat selection order 3). This places much weight on the availability domain, which agrees with other findings about habitat‐selection analyses (Mysterud and Ims 1998; Northrup et al. 2013). This pattern was even stronger for wRSA, where we found huge variability in the estimated coefficients at large sampling intervals, but no statistical significance, indicating that the information content in the data was insufficient. As the sampling interval increases, results for wRSA indicated the right coefficient sign and also found significant results as expected. The trend was similar for all three autocorrelation levels, but not as strong for very high autocorrelation. Possibly, wRSA would require even more data here.

When applying RSA to our empirical study case, we found that, similar to the simulation study, results from RSA varied strongly across individuals than results from SSA, although the pattern was less pronounced. Results from the empirical data are more difficult to interpret, as different individuals reflect both different landscape settings (each having their home range in a different part of the study area) and possibly different behavior and habitat preferences between individuals (Stuber et al. 2022). Parameter estimates for one individual (WB5) even switched sign for the habitat variable “imperviousness”, i.e., changed from avoiding built‐up areas to preferring them. This was likely due to a negative correlation with “habitat type”, but a positive correlation with “distance to water” (Figure S7). Wild boar prefer being close to water in the impenetrable reed beds and within forest habitat; if these conditions are provided, they even tolerate higher levels of human disturbance (Stillfried et al. 2017). WB5 was located in the Grunewald, both close to water where it rested regularly (river Havel and lake Wannsee area), but also close to housing areas (Figure 2 and Figure S11). Depending on which locations were filtered in the low temporal resolution scenario (here: 24 h), locations close to settlements were then overrepresented in the RSA. Likewise, rural individuals WB2 and WB7 strongly avoided impervious areas (Figure S11), whereby the overly strong selection coefficient in RSA is most probably due to the artificial boundaries of the background points that included impervious areas. Our finding of sign switching in parameter estimates corroborates previous research showing that habitat selection can differ depending on the time of day (e.g., for Florida panther, *Puma concolor coryi*, Onorato et al. 2011; for elk, *Cervus canadensis* , Roberts et al. 2017); however, we go beyond these studies by investigating our methods' behaviors at much finer temporal resolutions. Similar to the recently accumulating evidence showing that the mean‐field approach to measuring habitat selection may be misleading because of the personality in animal spatial use (Stuber et al. 2022; Hertel et al. 2020), our findings caution against blind comparison of habitat selection coefficients coming from the studies with different temporal resolutions.

Several recent studies review the methods used for habitat selection (e.g., Northrup et al. 2022; Fieberg et al. 2021), in an attempt to outline a coherent framework and to explain it to practitioners. We hope to further contribute to these efforts by assessing the sensitivity of two commonly used methods, RSA and iSSA, and a recently introduced wRSA, to the changes in sampling interval and the effect of landscape autocorrelation. Such an assessment is particularly valuable since biologging data is collected at increasingly finer temporal resolution (Alston et al. 2022). Our results corroborate the importance of sampling rates on estimated selection coefficients (e.g., Nisi et al. 2022). We expect our findings to further raise awareness of pitfalls underlying the comparison of selection coefficients obtained in different studies, to assist movement ecologists in choosing the appropriate method for habitat‐selection analysis, and to encourage further studies to investigate approaches that can remedy or counteract the sensitivities of habitat‐selection analyses.

## 5 Guidance for Practitioners and Outlook

Sampling interval and landscape configuration can significantly impact habitat selection coefficients. While we can change the sampling interval, there is usually relatively little we can do about the autocorrelation of the landscape the animals live in. Generally, we found that RSA and iSSA were slightly sensitive to the sampling interval but very sensitive to landscape configuration. The variability in estimates increased for all three methods with the degree of autocorrelation in the landscape. If there was no spatial autocorrelation, we found little to no effect of sampling interval or method used on habitat selection estimates. For highly correlated landscapes, like elevational gradients, we conclude that the variability of estimates only stabilizes at larger sampling intervals. As guidance, we suggest—if temporal sampling is coarse—both methods RSA and iSSA performed similarly.

As more and more fine‐scale tracking data sets become available, we expect that the issue of sampling interval will receive more attention, and more methodological improvements can be addressed. These include characterizing sampling intervals in terms of positional and velocity autocorrelation for different species (habitats) using continuous‐time movement models (Fleming et al. 2014; Noonan et al. 2019; Wang et al. 2019; Alston et al. 2022). wRSA is an attractive method that frees the analyst to determine availability and a manual subsampling of data, but comes at high computational costs (fitting a wRSA can take several orders of magnitude longer than an RSA or iSSA). High‐resolution data will also allow for new ways in characterizing behavioral states and state‐switching dynamics that can be directly integrated into SSA to understand fine‐scale habitat selection better (e.g., Klappstein et al. 2023; Pohle et al. 2024).

## Author Contributions

**Johannes Signer:** conceptualization (equal), formal analysis (equal), methodology (equal), validation (equal), writing – original draft (equal). **Cédric Scherer:** data curation (equal), formal analysis (equal), methodology (equal), visualization (equal). **Viktoriia Radchuk:** writing – review and editing (lead). **Carolin Scholz:** investigation (equal), writing – review and editing (equal). **Florian Jeltsch:** project administration (equal), writing – review and editing (equal). **Stephanie Kramer‐Schadt:** conceptualization (equal), methodology (equal), validation (equal), writing – original draft (equal).

## Conflicts of Interest

The authors declare no conflicts of interest.

## Supporting information


Data S1

## Data Availability

All data, simulated and empirical, as well as the code is reproducibly provided in the department's official GitHub repository [https://github.com/EcoDynIZW/Signer_2025_EcolEvol]. The repository is also archived at Zenodo (10.5281/zenodo.15376245). The empirical data is also available on movebank.org.
