# Supplementary material for: The 4th Dimension in Animal Movement: The Effect of Temporal Resolution and Landscape Configuration in Habitat‐Selection Analyses
Source: Ecol Evol. 2025 May 12;15(5):e71434. doi: 10.1002/ece3.71434 (PMC12068901; doi:10.1002/ece3.71434)
Supplement: Supplementary file 1 — Data S1 [file ECE3-15-e71434-s001.docx]

# Supporting information

**Article “The 4th dimension in animal movement: The effect of temporal resolution and landscape configuration in habitat selection analyses”**

Johannes Signer, Cédric Scherer, Viktoriia Radchuk, Carolin Scholz, Florian Jeltsch, Stephanie Kramer-Schadt

## Supporting information I: Simulation study

### Simulated landscape sets


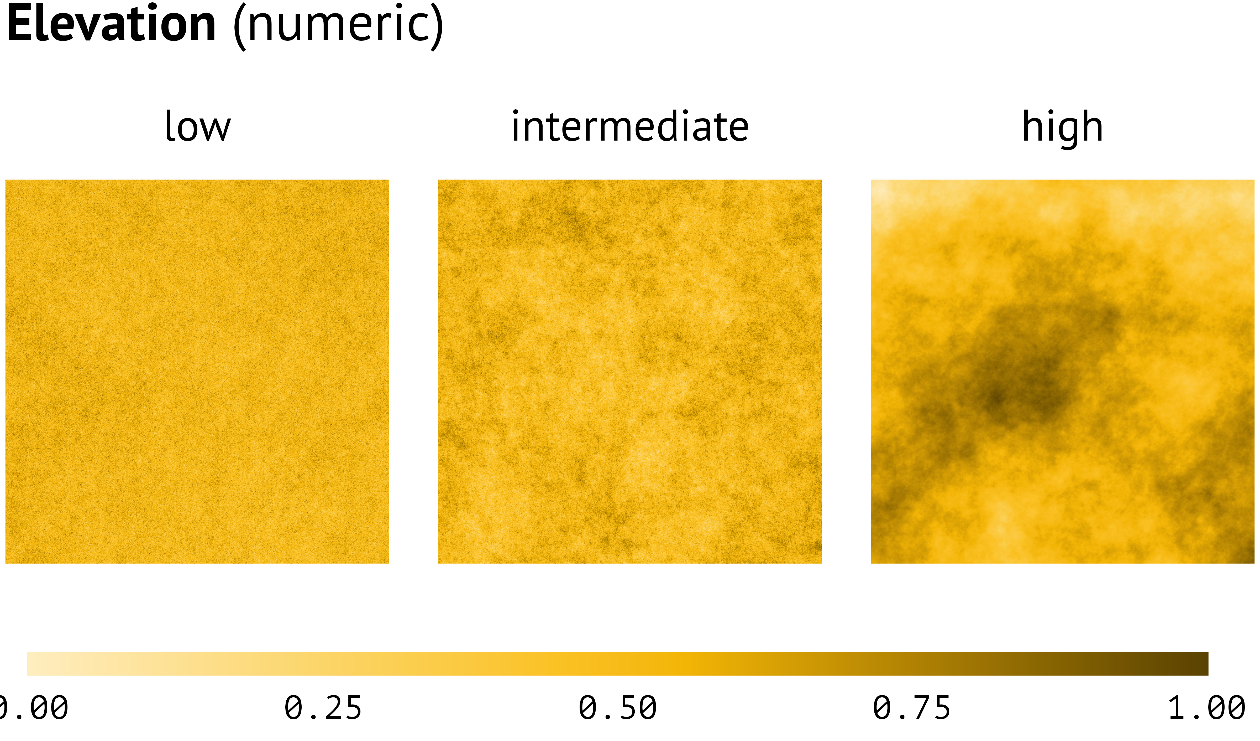


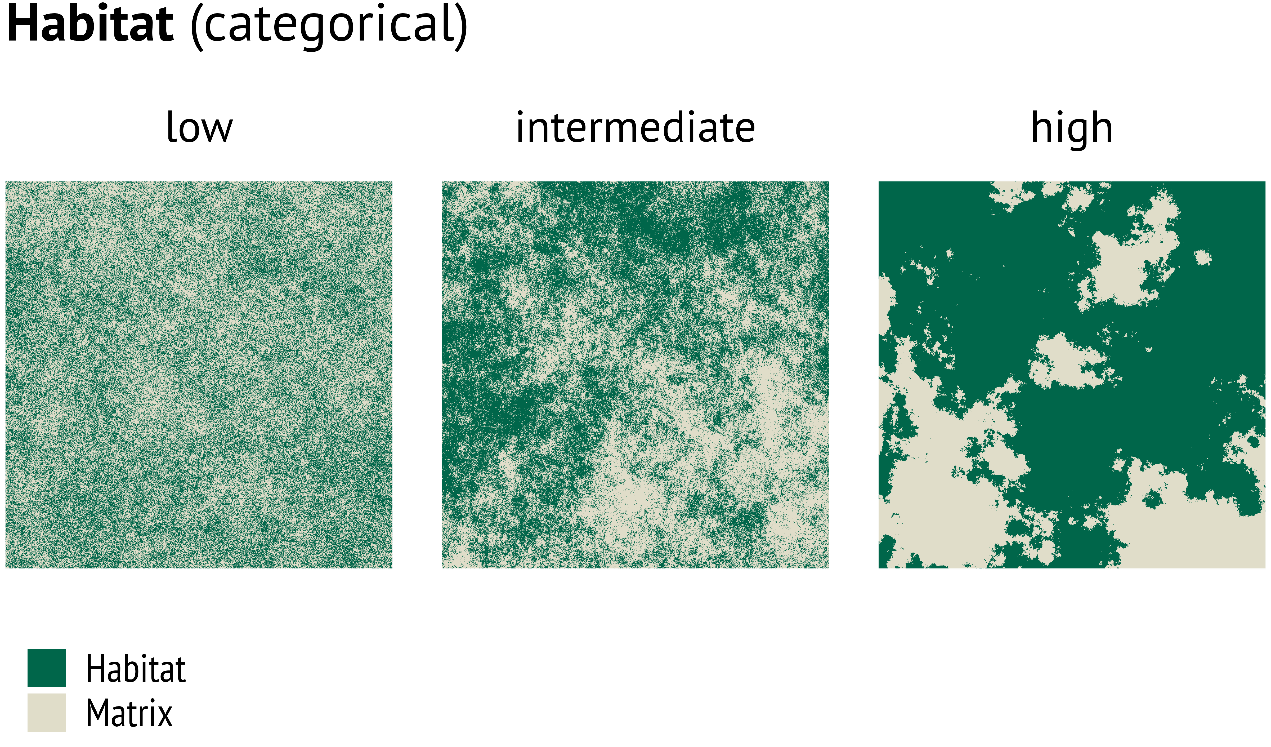


### Fig S1. Environmental predictor variables based on neutral landscape simulators (see main text for details). Examples of simulated landscape with, low, intermediate and high autocorrelation. Movement simulations were run on all six landscapes (each landscape simulation repeated ten times) as single variables and also on five combinations: elevation and habitat combined as low, medium and high each, and a combination of elevation with low autocorrelation with habitat as a high one and vice versa (see Fig. 5 in main manuscript).

### Multicollinearity plots


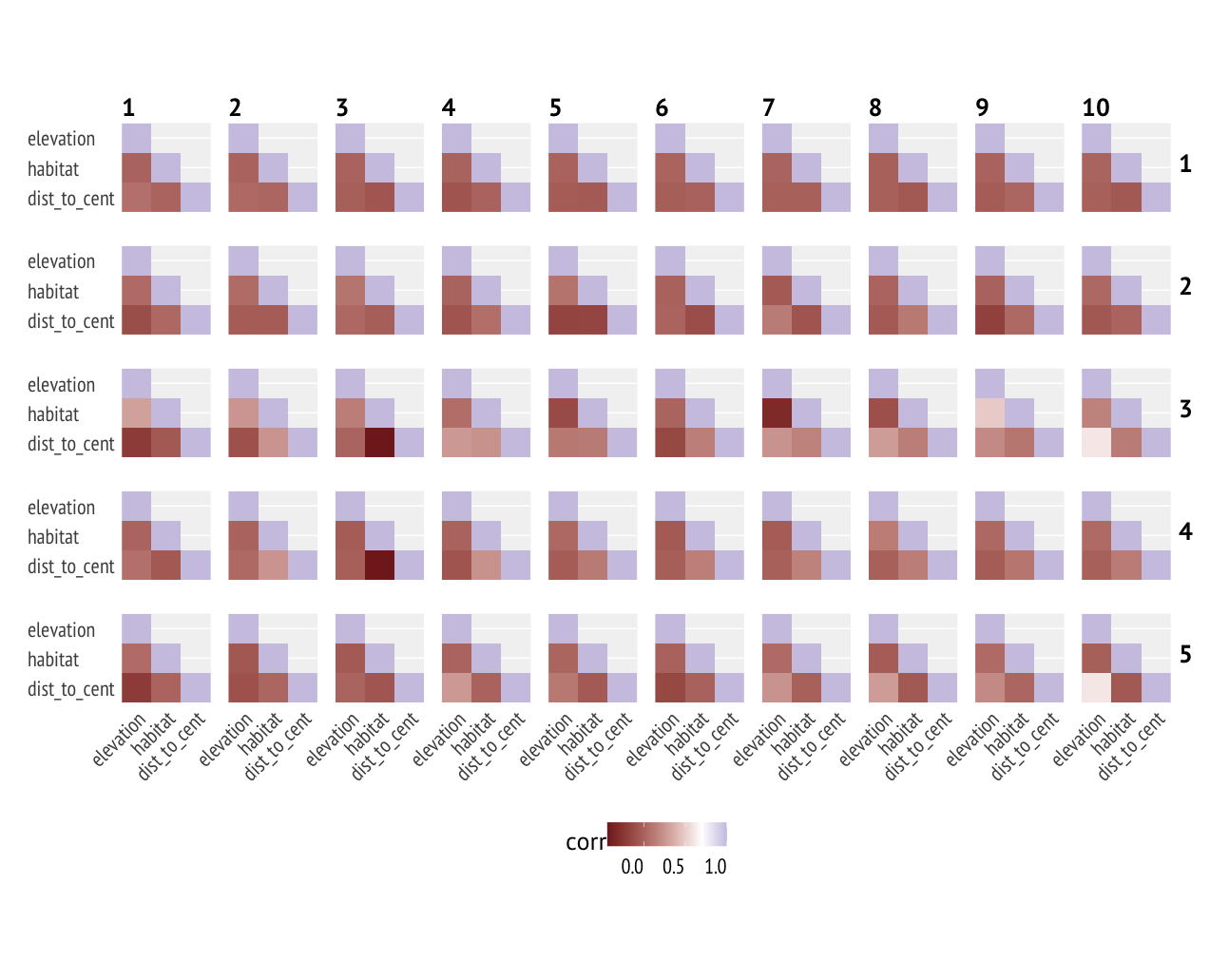


Fig S2. Visualization of correlation matrices for all landscape variables (Fig. S1) that were used to simulate movement tracks. Correlations were computed as Spearman’s rank correlation coefficients. Note that distance to home range centre (Distance home range) was used as an auxiliary variable to simulate realistic, home-ranging movement patterns restricted to a home range, but this variable was not included in the model fits, because HR centres are typically not known. All landscapes showed some correlations between variables. Of highest concern are correlations with “Distance home range” that cause conflicting selection behaviour. Columns (1 – 10 are different landscape realizations), rows (1 – 5) indicate different levels of landscape autocorrelation. 1 – 3 indicate low, intermediate and high landscape autocorrelation for both covariates (elevation and habitat). 4 indicates low autocorrelation of the landscape for elevation and high correlation for habitat. Finally, 5 indicates high correlation for elevation and low correlation for habitat. Distance to center was always the same.

### Estimates for all methods


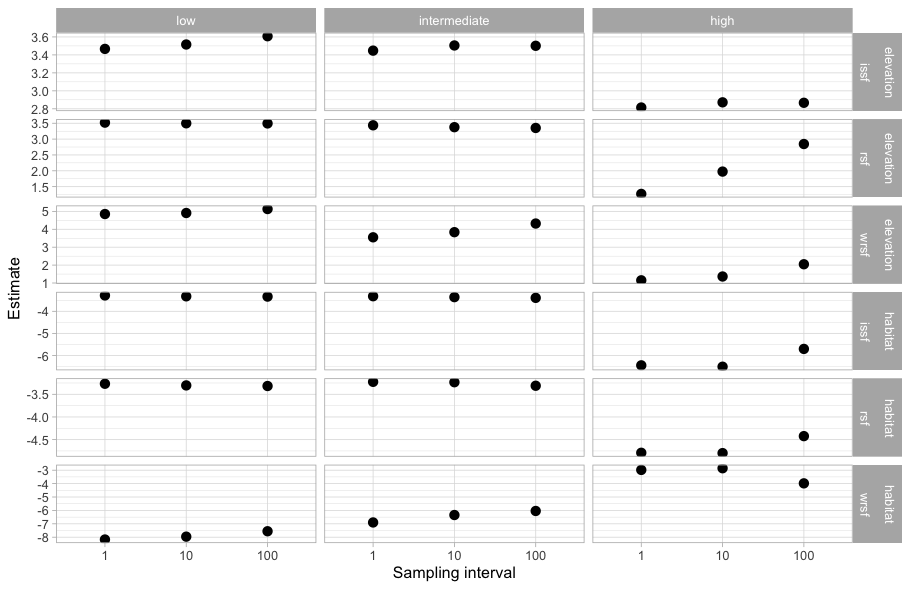


Fig S3. Estimated selection parameters for increasing subsampling interval resulting from the tested methods, using different simulated landscapes. Here we show the results for single landscape (i.e., all only one variable was used for simulation and model fitting). Different levels of landscape autocorrelation are shown in different columns. We show the mean of the fitted estimate.


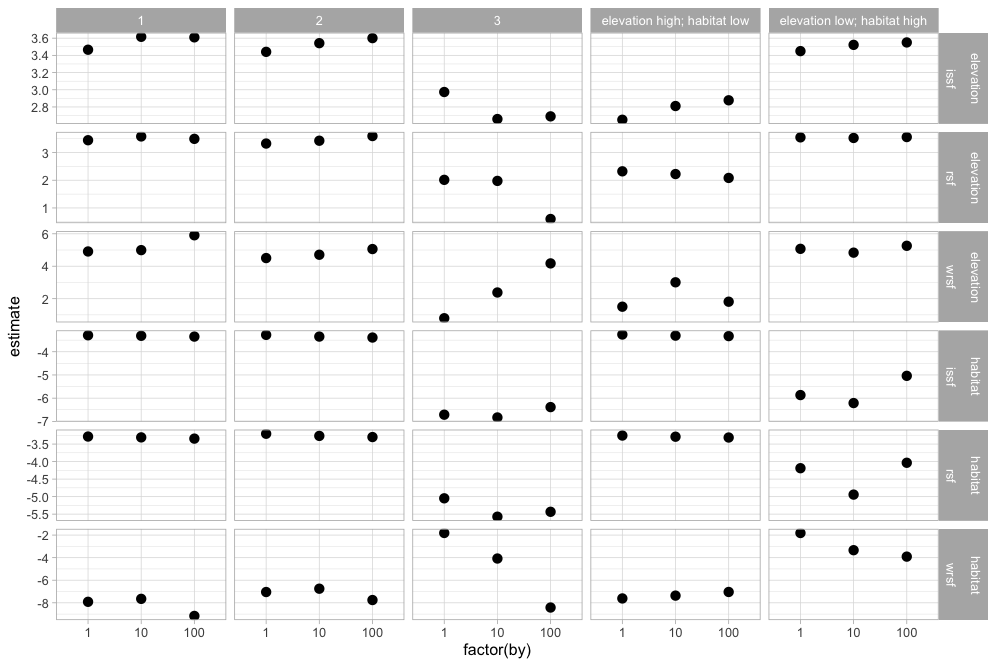


Fig S4 – Estimated selection parameters for increasing subsampling interval resulting from the tested methods, using different simulated landscapes. Columns show different autocorrelation levels of the landscape. Columns 1,2 and 3 indicate that both variables had low, intermediate or high landscape correlation. The last two columns show the results for scenarios where one variable had a low and the other a high level of autocorrelation. Here we show the results for combined landscape (i.e., both variables were used for simulation and model fitting). We show the mean of the fitted estimate.

### Single landscape selection coefficients without home ranging


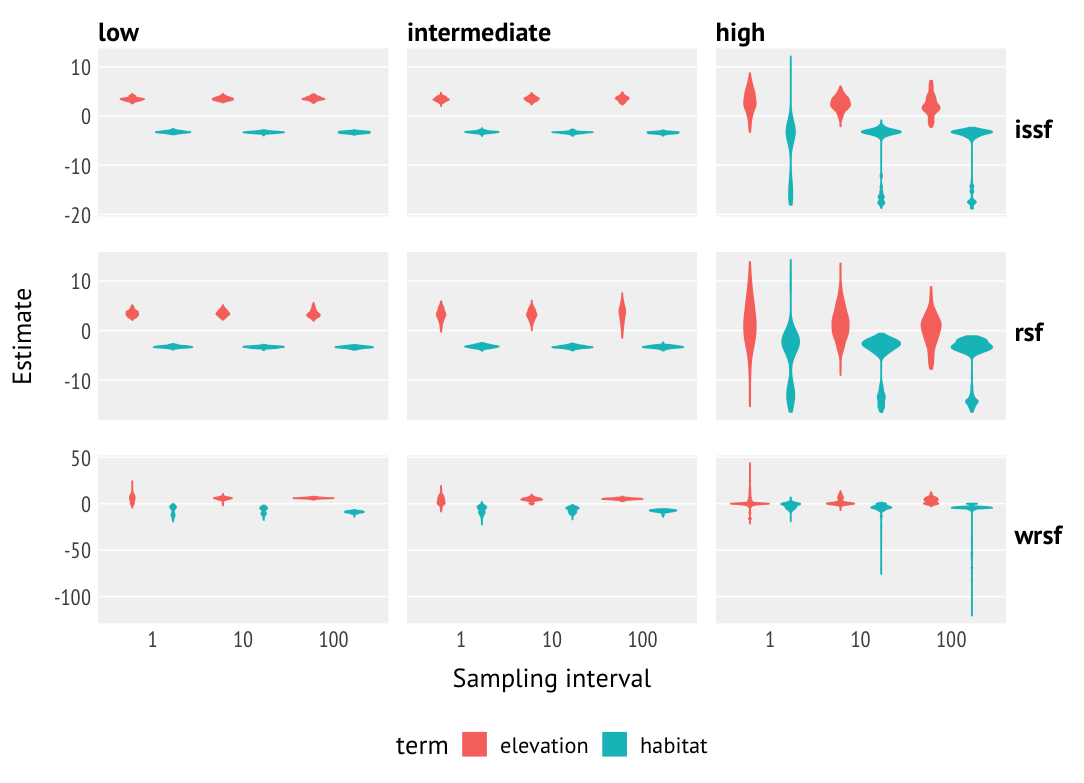


Fig S5 Same figure as Fig. 3 in the manuscript, but without home-ranging in simulation. The selection coefficient for distance to center was set to 0 during the simulations.

## Supporting information II: Empirical case study

### Environmental predictor variables


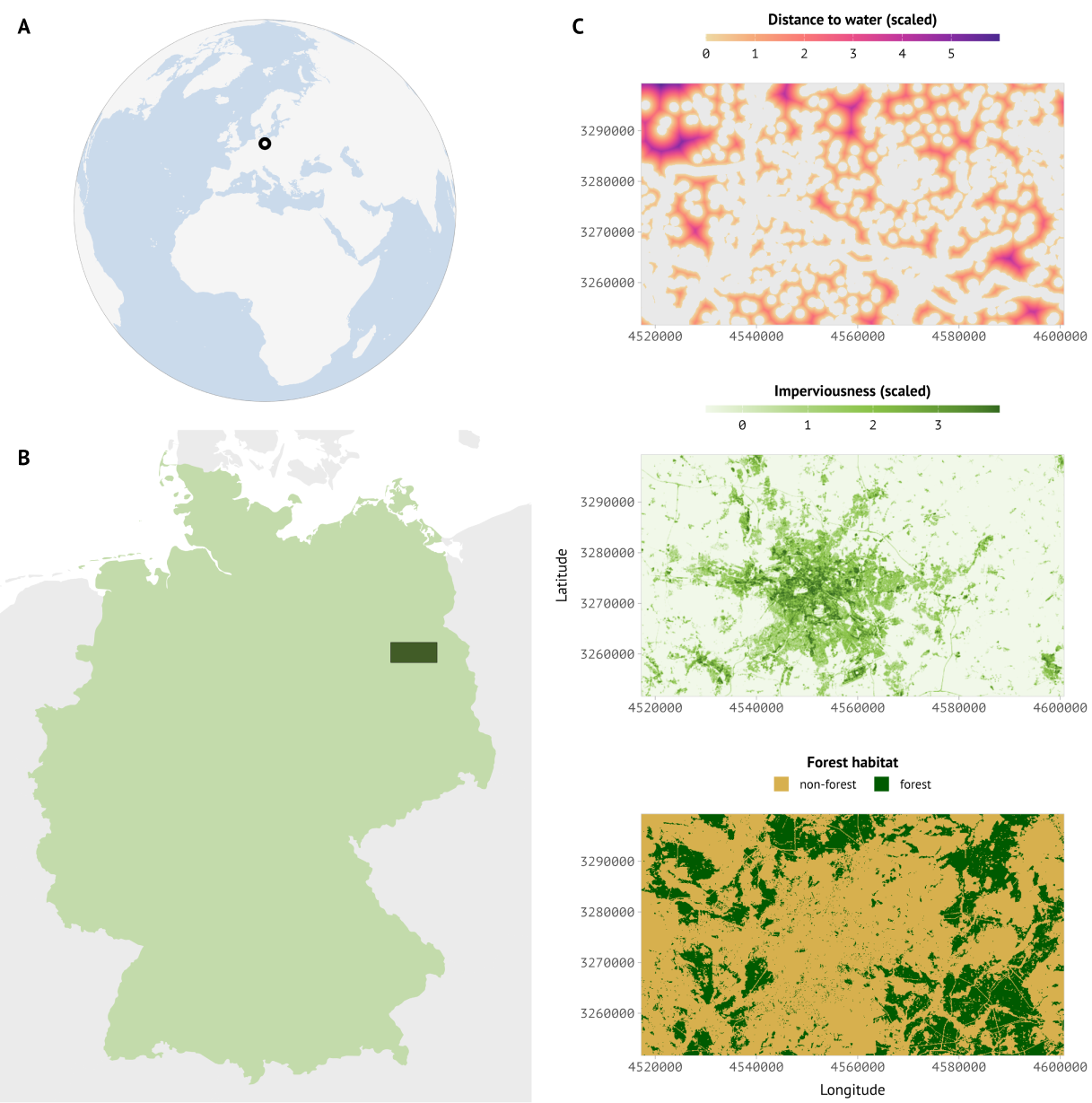


Fig. S6: Study area and gradients of the three environmental variables ‘distance to water’, ‘imperviousness’ and ‘forest habitat’ (binary).


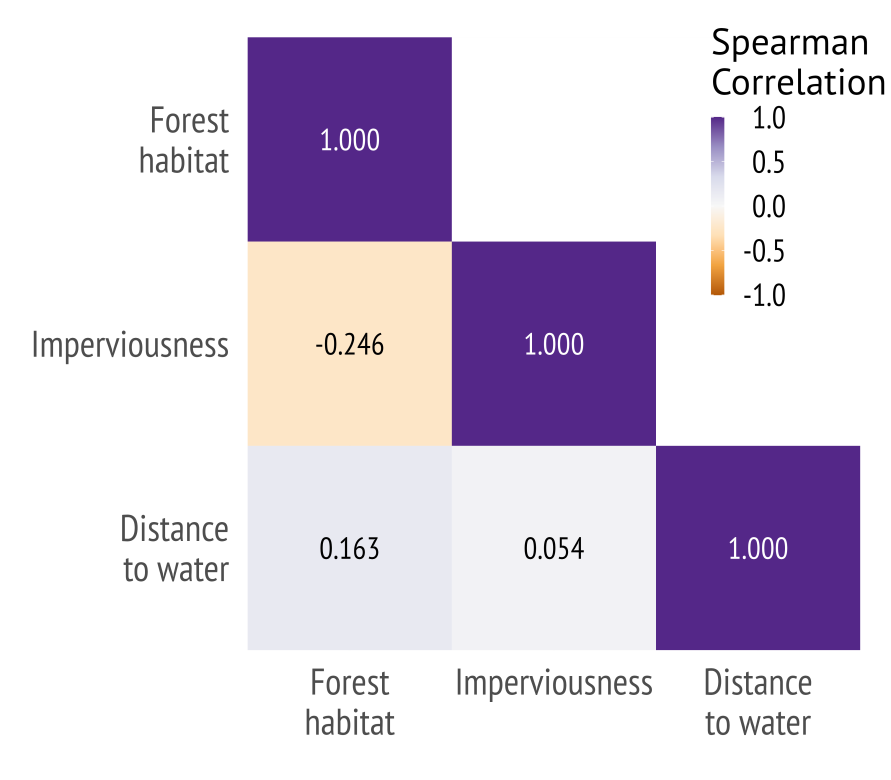


Fig S7. Visualization of correlation matrices for all three landscape variables that were used to analyse the wild boar movement tracks. Correlations were computed as Spearman’s rank correlation coefficients.

### Movement data

Table S1: Number of locations for the selected wild boar for the highest (30 min) and lowest resolution (24 hrs).

| ID | # Locations  (30 min resolution) | # Locations  (1440 min resolution) |
| --- | --- | --- |
| 1 | 7,366 | 153 |
| 2 | 10,028 | 209 |
| 4 | 11,499 | 240 |
| 5 | 9,187 | 192 |
| 6 | 9,458 | 198 |
| 7 | 13,861 | 289 |
| 8 | 8,749 | 182 |
| 9 | 8,701 | 181 |


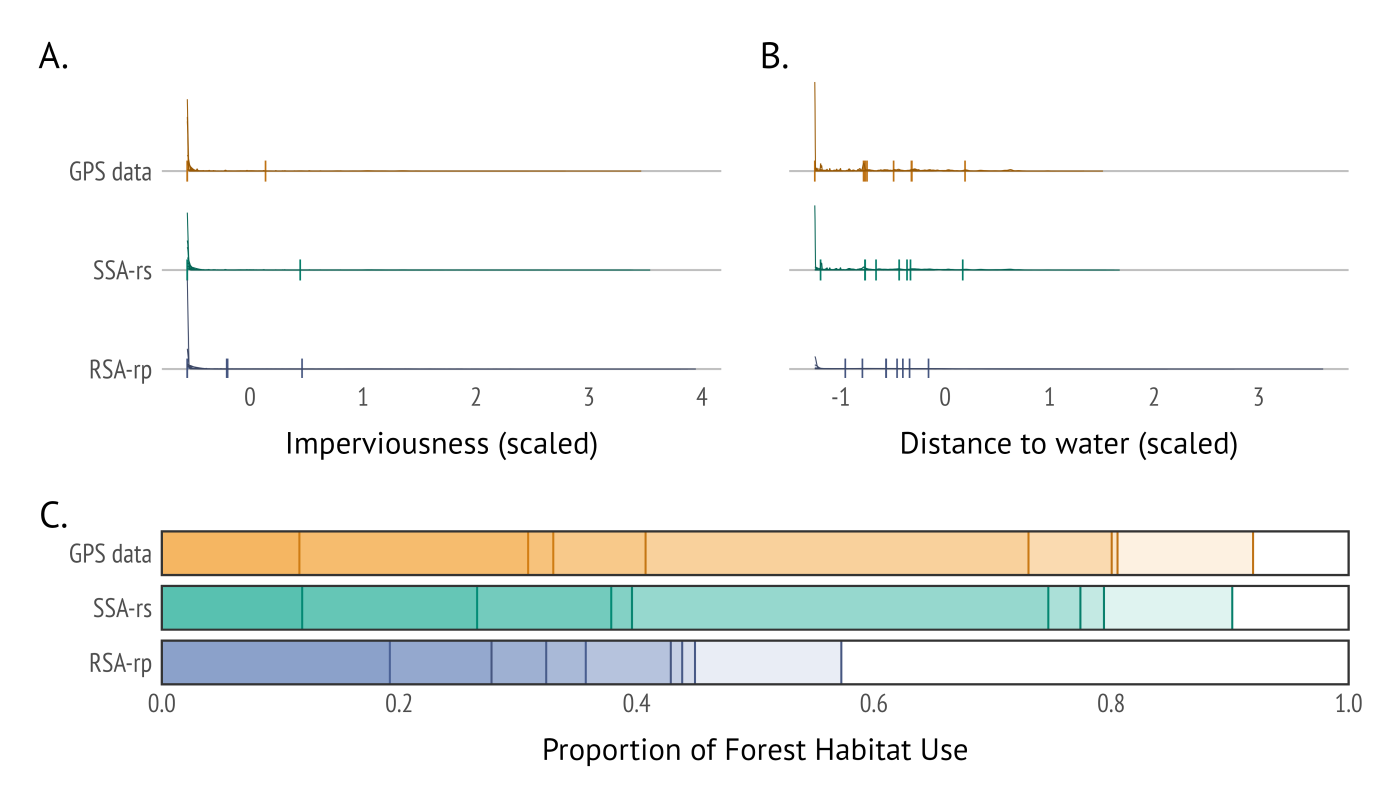


30 min interval

*Fig. S8: Distributions of the environmental variables for each wild boar for the locations of the recorded movement track (“sampled positions”) and the sampled available locations in the different habitat selection methods (SSA, RSA). Note, that wRSA does not appear here because the parameter estimation procedure does not require any sampling of available locations. The vertical ticks indicate the average value per environmental variable and animal. The data shows the sampling interval with the highest resolution (30 minutes).*

6 hrs interval


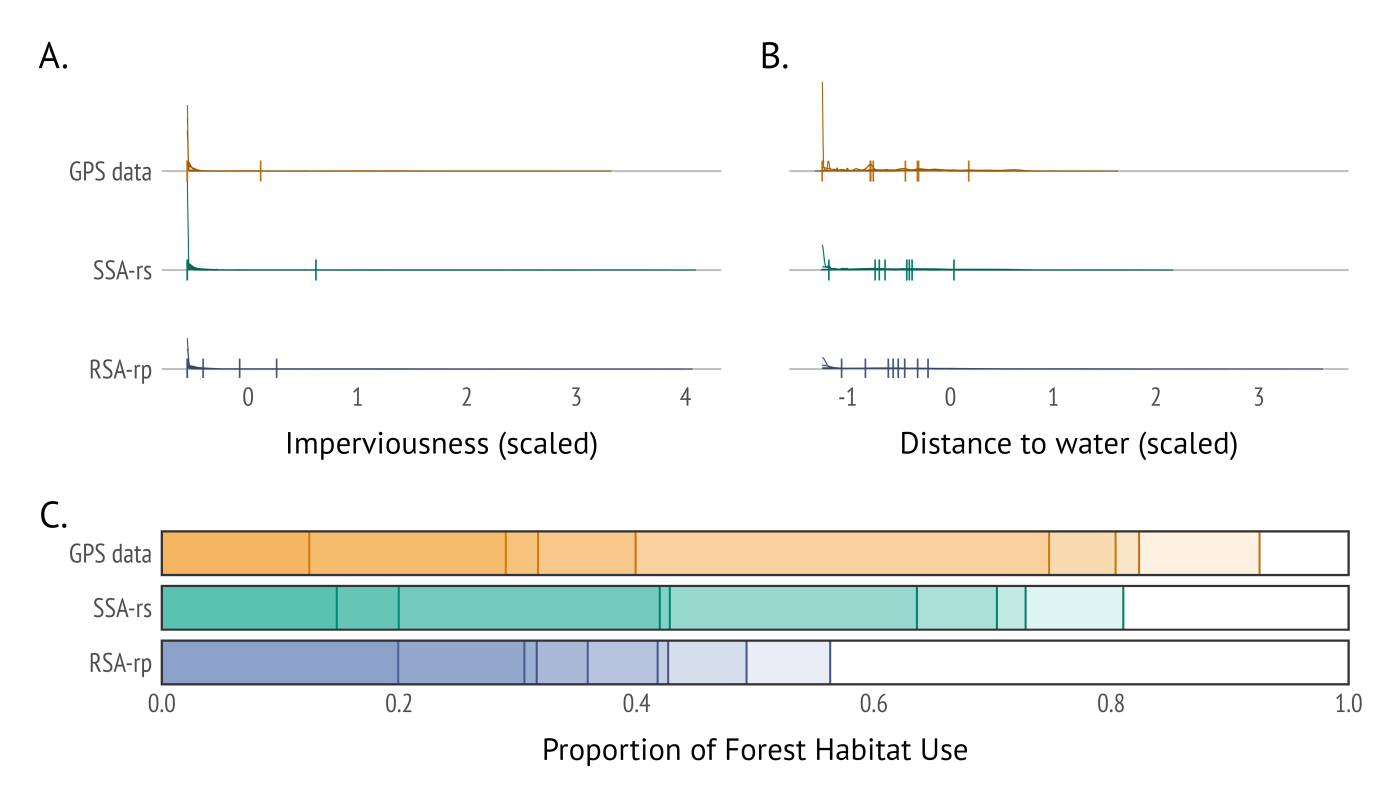


*Fig. S9: Distributions of the environmental variables for each wild boar for the locations of the recorded movement track (“sampled positions”) and the sampled available locations in the different habitat selection methods (SSA, RSA). Note, that wRSA does not appear here because the parameter estimation procedure does not require any sampling of available locations. The vertical ticks indicate the average value per environmental variable and animal. The data shows the sampling interval with an intermediate resolution (360 minutes ~ 6 hours).*

24 hrs interval


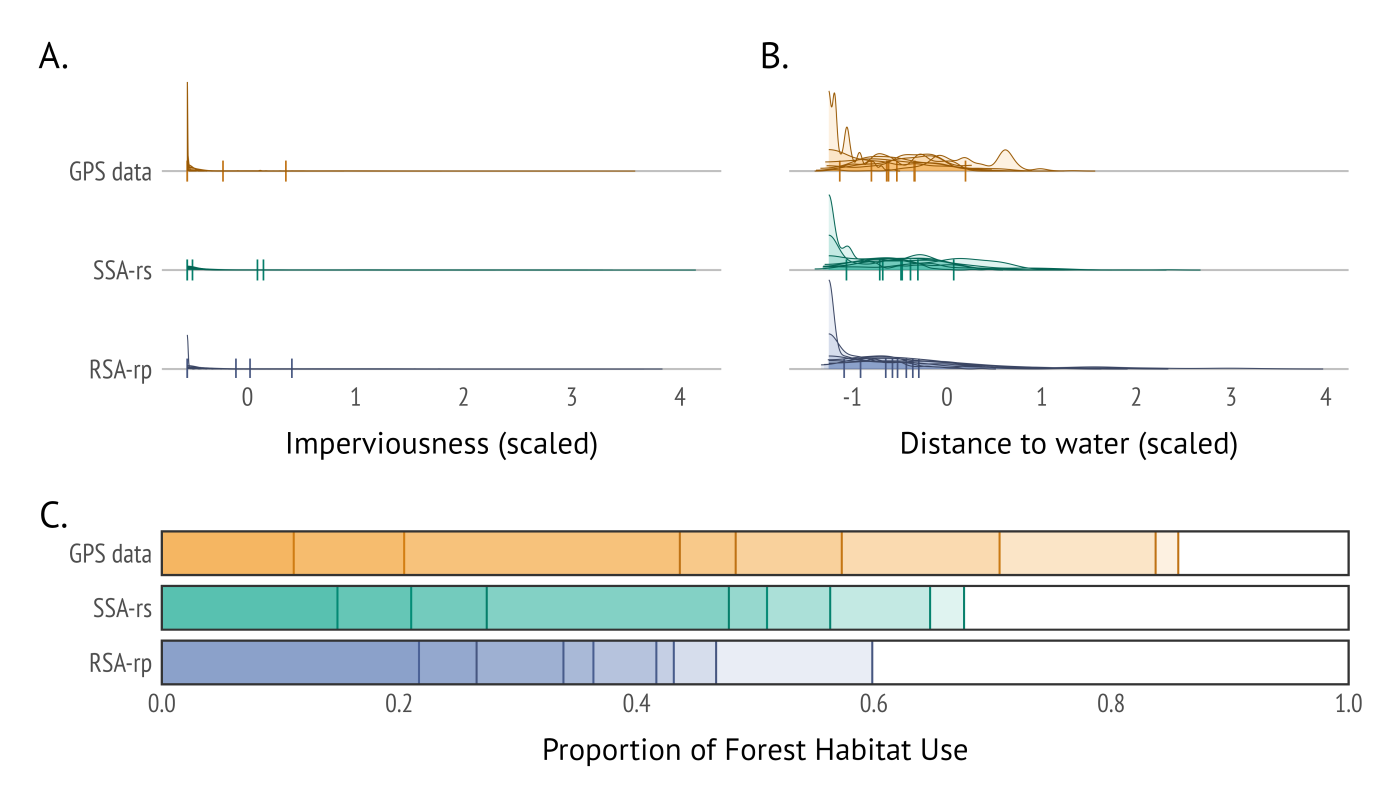
*Fig. S10: Distributions of the environmental variables for each wild boar for the locations of the recorded movement track (“sampled positions”) and the sampled available locations in the different habitat selection methods (SSA, RSA). Note, that wRSA does not appear here because the parameter estimation procedure does not require any sampling of available locations. The vertical ticks indicate the average value per environmental variable and animal. The data shows the sampling interval with the coarsest resolution (1440 minutes ~ 24 hours).*


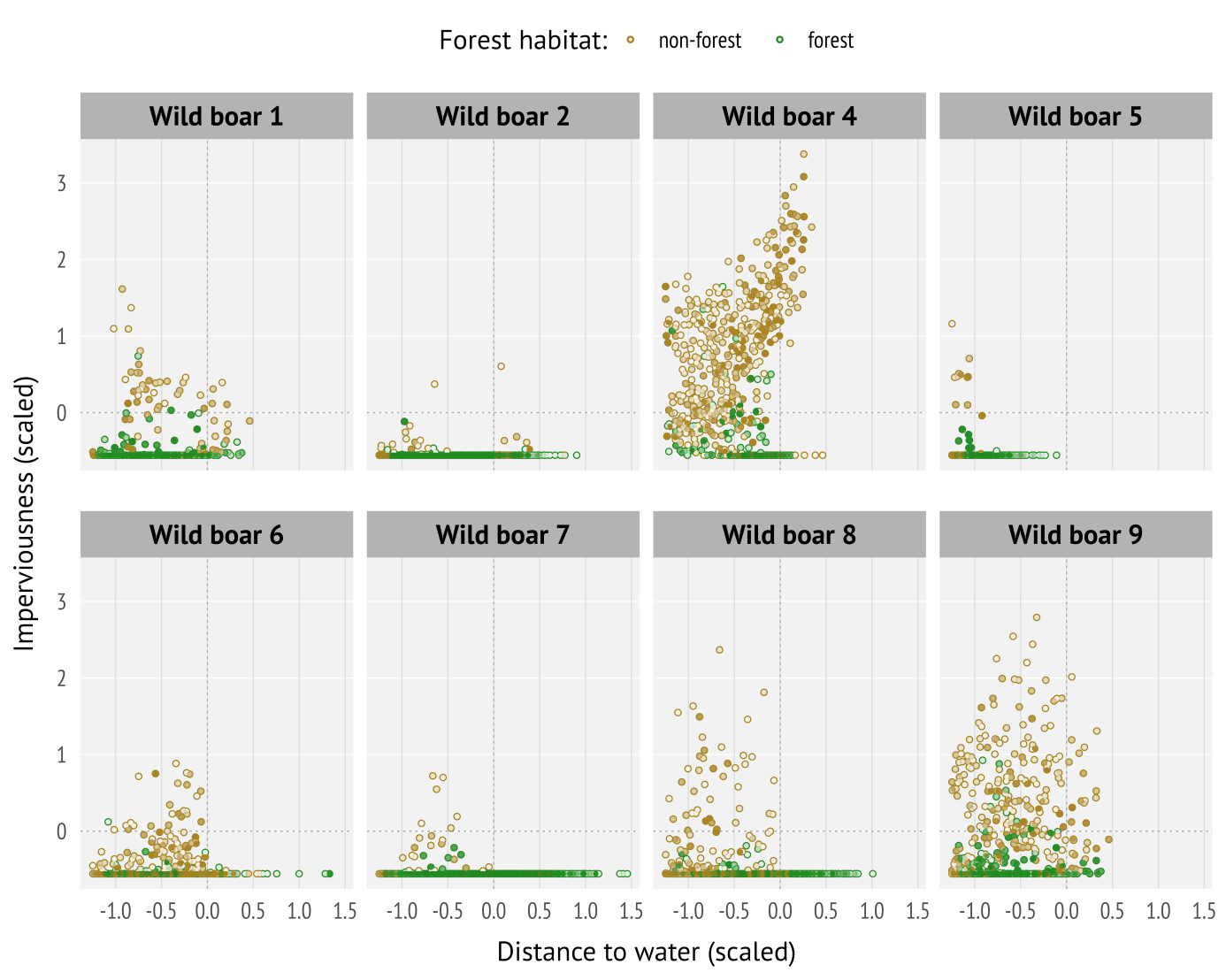


*Fig. S11: Distribution of the selected environmental variables for the real wild boar (WB) tracks. The numbering corresponds with their original ID.*
